# A Rare Lesion of the Thoracic Wall: Giant Scapulothoracic Bursitis

**DOI:** 10.7759/cureus.30113

**Published:** 2022-10-09

**Authors:** Selcuk Gurz, Necmiye Temel, Asli Tanrivermis Sayit, Yurdanur Sullu

**Affiliations:** 1 Department of Thoracic Surgery, Ondokuz Mayis University Faculty of Medicine, Samsun, TUR; 2 Department of Radiology, Ondokuz Mayis University Faculty of Medicine, Samsun, TUR; 3 Department of Pathology, Ondokuz Mayis University Faculty of Medicine, Samsun, TUR

**Keywords:** giant cyst, general thoracic surgery, scapulothoracic, cyst, bursitis

## Abstract

Scapulothoracic bursitis, a rare lesion of the thoracic wall, usually presents as a cystic mass growing at the scapulothoracic interface. Histopathologically, it is characterized by the presence of synovial cells lining the interior of the thickened fibrotic cystic wall and capillary proliferation. A 48-year-old male patient was admitted to our clinic with a complaint of swelling in the back. The magnetic resonance imaging of the lung and mediastinum showed a 43 mm × 130 mm axial lesion in the left infrascapular area between the external muscles and the serratus anterior muscle, hyperintense on T2 sequence, not suppressed on fat-suppressed sequences, with a peripheral minimally contrasted septated collection area. The patient underwent surgical total excision and was discharged on the second postoperative day with no morbidity. Histopathology of the tissue was reported as soft tissue compatible with an inflamed cyst wall with prominent fibroblastic proliferation. Scapulothoracic bursitis lesions can be treated with non-invasive or minimally invasive methods. However, when it becomes a giant lesion occupying space on the thoracic wall and has hemorrhagic content, surgical excision is the treatment of choice.

## Introduction

Scapulothoracic bursitis, a rare lesion of the thoracic wall, usually occurs as inflammation of the bursa as a result of mechanical stress, sports, or trauma [1]. Movement of the scapula with vigorous arm movements causes swelling of the back without pain or redness. Due to the swelling of the bursae, crepitus may be heard with movement in some patients [2]. Magnetic resonance imaging of the thorax is the best imaging tool for soft tissue pathology [2]. Treatment of scapulothoracic bursitis can be conservative or surgical. In cases where conservative treatment is inadequate, surgical excision is the definitive treatment method [3]. After surgical excision, it is characterized by the presence of synovial cells lining the interior of the thickened fibrotic cystic wall and capillary proliferation [2]. In this case report, we aimed to present a rare case of giant scapulothoracic bursitis.

## Case presentation

**Figure 1:**
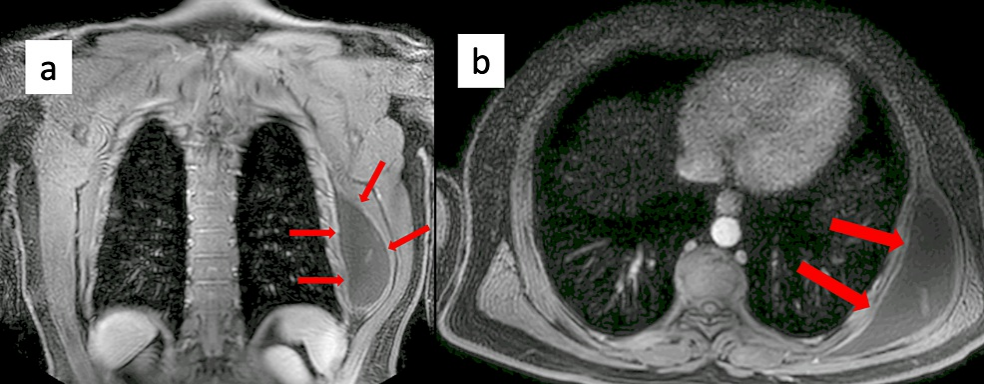
(a) Coronal section and (b) axial section images of giant scapulothoracic bursitis on MRI of thorax.

A 48-year-old man noticed swelling in his back one week ago and had difficulty moving his arm. Thorax tomography was performed on the patient who applied with the complaint of increasing swelling in his back. A smooth circumscribed hypodense space-occupying lesion measuring approximately 15 cm × 4.5 cm × 13.5 cm extending inferiorly along the posterolateral wall of the thorax starting from the anterior left scapula. The lesion was fluctuating on palpation and a hemorrhagic fluid was aspirated on puncture. The patient was admitted to Ondokuz Mayıs University Faculty of Medicine, Department of Thoracic Surgery, for further examination and treatment. The magnetic resonance imaging of the thorax showed a 43 mm × 130 mm axial lesion between the external muscles and serratus anterior muscle in the left infrascapular area, hyperintense in T2 sequence, not suppressed in fat-suppressed sequences, with a septated collection area with minimal peripheral contrast (Figure 1).

**Figure 2:**
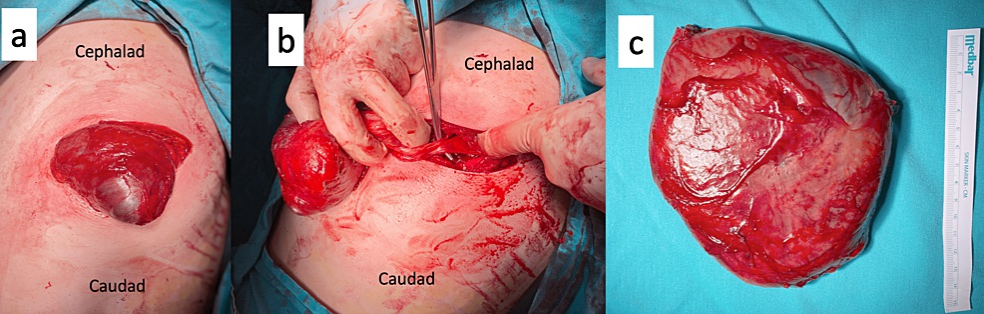
The giant cyst was (a)-separated from the surrounding tissues and delivered as encapsulated, (b)-capsular stalk was ligaturised and (c)-totally excised.

Single-lumen intubation was performed under general anesthesia. In the right lateral decubitus position, subcutaneous fatty tissues were crossed with a 10 cm skin incision made from the inferior left scapula. The giant cystic lesion was reached by dissecting the latissimus dorsi and serratus anterior muscles in the direction of the muscle fibers. The lesion was delivered by separating the capsule from the surrounding tissues with blunt and sharp dissection. The lesion stalk was excised with ligasure (Figure 2).

**Figure 3:**
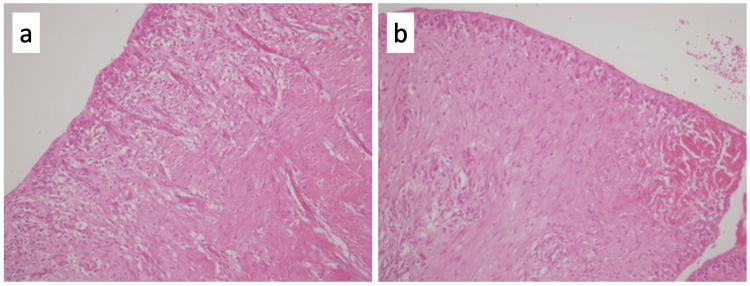
Cyst wall composed of collagenized fibrous tissue containing vascular structures and inflammatory cells H-E ×200 (a, b).

One hemovac drain was placed between the tissues. The tissues were closed duly. The patient was discharged on the second postoperative day with no morbidity. Histopathology of the tissue was reported as soft tissue compatible with an inflamed cyst wall containing prominent fibroblastic proliferation (Figure 3).

## Discussion

Scapulothoracic bursitis is a lesion caused by abnormal biomechanics between the scapula and the thoracic wall. It can occur posttraumatically, idiopathically, or as a result of thoracic wall surgery [4,5].

Kuhn et al. defined two major and four minor bursae in the scapulothoracic joint [6]. Major bursae are located between the serratus anterior muscle and the thoracic wall (scapulothoracic bursa) and between the serratus anterior muscle and the subscapular muscle (subscapularis bursa). In their study on eight cadavers [1], Williams et al. divided the scapulothoracic area into three groups as superficial, intermediate, and deep. The scapulothoracic and subscapular bursae are deep; the scapulotrapezial bursa (between the superomedial scapula and the trapezius muscle) is intermediate; and the inferior scapular bursa (between the inferior scapular angle and the latissimus dorsi muscle) is superficial.

Bursitis occurs with bursa inflammation after mechanical stress, sports, or trauma [1]. Scapulothoracic bursitis manifests as pain and non-fluffy swelling that increases with activity in patients. The diagnosis is made by tomography and magnetic resonance imaging. The lesion is usually seen as a well-circumscribed cystic lesion between the serratus anterior and the thoracic wall [7-10]. In our case, the lesion was in the deep group between the thoracic wall and the serratus muscle. There was no redness and no heat increase. The patient had no history of trauma and was a construction worker.

The first option in the treatment of scapulothoracic bursitis is systemic non-steroidal anti-inflammatory drugs, physiotherapy, local steroid, and analgesia injection [11]. Surgical treatment is recommended for patients whose complaints do not improve despite medical treatment. Partial scapulectomy, resection of the superomedial angle, and arthroscopic techniques are recommended methods [3].

Scapulothoracic bursitis lesions can be treated with non-invasive or minimally invasive methods. However, when it becomes a giant lesion occupying space on the thoracic wall and has hemorrhagic content, surgical excision is the treatment of choice.

## Conclusions

In conclusion, although local treatments are recommended in cases of scapulothoracic bursitis, surgical excision should be performed in large lesions. In the differential diagnosis, elastofibroma, abscess, hematoma, and sarcomas such as malignant fibrous histioma, liposarcoma, and lymphangioma should be kept in mind.

## References

[1] Williams GR Jr, Shakil M, Klimkiewicz J, Iannotti JP (1999). Anatomy of the scapulothoracic articulation. Clin Orthop Relat Res.

[2] Dzian A, Skaličanová M, Fučela I, Malík M, Mičák J (2019). Bilateral cystic lesions of the chest wall: presentation of scapulothoracic bursitis. Int J Surg Case Rep.

[3] Warth RJ, Spiegl UJ, Millett PJ (2015). Scapulothoracic bursitis and snapping scapula syndrome: a critical review of current evidence. Am J Sports Med.

[4] Fujikawa A, Oshika Y, Tamura T, Naoi Y (2004). Chronic scapulothoracic bursitis associated with thoracoplasty. AJR Am J Roentgenol.

[5] Lehtinen JT, Tetreault P, Warner JJ (2003). Arthroscopic management of painful and stiff scapulothoracic articulation. Arthroscopy.

[6] Kuhn JE, Plancher KD, Hawkins RJ (1998). Symptomatic scapulothoracic crepitus and bursitis. J Am Acad Orthop Surg.

[7] Higuchi T, Ogose A, Hotta T (2004). Clinical and imaging features of distended scapulothoracic bursitis: spontaneously regressed pseudotumoral lesion. J Comput Assist Tomogr.

[8] Griffiths HJ, Thompson RC Jr, Galloway HR, Everson LI, Suh JS (1991). Bursitis in association with solitary osteochondromas presenting as mass lesions. Skeletal Radiol.

[9] El-Khoury GY, Bassett GS (1979). Symptomatic bursa formation with osteochondromas. AJR Am J Roentgenol.

[10] Sisto DJ, Jobe FW (1986). The operative treatment of scapulothoracic bursitis in professional pitchers. Am J Sports Med.

[11] Conduah AH, Baker CL 3rd, Baker CL Jr (2010). Clinical management of scapulothoracic bursitis and the snapping scapula. Sports Health.

